# Effect of secondary phase on the electromagnetic shielding effectiveness of magnesium alloy

**DOI:** 10.1038/s41598-018-19933-7

**Published:** 2018-01-26

**Authors:** Shangyu Gao, Xianhua Chen, Fusheng Pan, Kai Song, Chaoyue Zhao, Lizi Liu, Xiaofang Liu, Di Zhao

**Affiliations:** 1 College of Materials Science and Engineering, Chongqing University, Chongqing, 400045 China; 2 National Engineering Research Center for Magnesium Alloys, Chongqing University, Chongqing, 400045 China; 3 Chongqing Academy of Science and Technology, Chongqing, 401123 China

## Abstract

The microstructure, electrical conductivity, and electromagnetic interference (EMI) shielding effectiveness (SE) of Mg-xZn and Mg-xSn (x = 3,5) alloys prepared under different rolling and heat treatment conditions were systematically investigated to understand the effect of secondary-phase orientation on the electromagnetic-shielding property of magnesium alloys. Alloys were rolled to form basal textures and then subjected to different durations of solid-solution treatment and aging to induce the precipitation of secondary-phase particles along a specific direction. Experimental results indicated that in Mg-Zn and Mg-Sn alloys, secondary phases precipitated along directions perpendicular and parallel to the basal plane, respectively. When the direction of the incident electromagnetic wave is perpendicular to the basal plane, precipitates in Mg-Sn alloy parallel to the basal plane improve SE. The increment in SE is mainly attributed to the improvement in the reflection and multiple reflection losses of incident electromagnetic waves, which are caused by increasing the amounts of precipitates with specific orientations. Mg-5Sn alloy subjected to 16 h of solution treatment at 480 °C and 60 h of artificial aging at 170 °C for 60 h exhibited the maximum value of 107–89 dB and maximum increment in SE of 13 dB at 1200 MHz.

## Introduction

Given the rapid development of electronic science and technology, electromagnetic radiation pollution has become the fifth most prevalent pollution after air, water, noise, and solid-waste pollution^1^. Therefore, electromagnetic interference (EMI) has become a matter of concern, especially in the field of 3 C and electrical apparatus. Research on the development of electromagnetic shielding materials has rapidly expanded, and most related studies have focused on the application of metallic materials, thin films, conductive polymers, and composite materials as electromagnetic shielding materials^2,3^. Polymer materials and composites have been extensively developed given their considerable weight and cost advantages. Nevertheless, polymer materials and composites have inferior electromagnetic shielding effectiveness (SE) given their lower electrical conductivity than metallic materials^4^. Moreover, the production of polymer materials for practical applications is limited by material wear, process complexity, high cost, and other issues^5–8^.

Metallic materials can be used as excellent electromagnetic shielding materials because of their good electrical conductivity and high permeability^4,9^. In particular, magnesium alloys, given their high conductivity, are a promising electromagnetic shielding material^10^. Compared with foam, coating, and composite materials, magnesium alloys have superior mechanical and electromagnetic shielding properties and can be used as engineering structural materials; compared with traditional metal materials, magnesium alloys have the advantages of low density, light weight, and high specific strength^11–13^.

We previously found that in the frequency range of 30–1500 MHz, the SEs of pure magnesium, AZ31, AZ61, ZK60, ZM61, and other commonly used magnesium alloys are significantly better than those of aluminum alloy with the same thickness but are lower than those of copper and copper alloys^14^. In our subsequent study, ZK60 magnesium alloy was treated through low-temperature aging, and its EMI shielding capacity was enhanced by inducing the precipitation of a secondary phase^15,16^. Song *et al*. reported that increasing Zn content decreases the EMI SE of cast and solutionized alloys^17^. Subsequent studies revealed that converting the random texture of the alloy to basal texture improves SE. If basal texture intensity continues to increase, SE also increases by small increments^18^. The results of the above studies indicated that the secondary phase influences the electromagnetic SE of magnesium alloys.

The secondary phase of magnesium alloys has been studied for many years, and previous studies have mainly focused on the mechanisms that underlie the influence of secondary phase on the mechanical properties, corrosion resistance, damping properties, and thermal conductivity of alloys^19–21^. Although magnesium alloys are potentially excellent EMI shielding materials, few reports exist on the influence of secondary phase on the EMI shielding properties of magnesium alloys^10^. In magnesium alloys, the content, morphology, type, and orientation of the secondary phases are highly complex and dependent on alloy type and processing technologies. The present study focuses on the effect of secondary-phase orientation on the electromagnetic shielding properties of magnesium alloys.

During aging, the secondary phase precipitates along a specific crystal plane. Thus, the orientation of the secondary phase can be controlled as long as grain orientation is controlled. Magnesium alloys can be rolled to obtain a strong texture with grains that are arranged along a specific orientation^22^. Moreover, the types of secondary phases formed in various types of alloys considerably vary in accordance with the elemental content of the alloy. Thus, if alloys with different components are rolled and then subjected to solution treatment and aging, alloys with secondary phases that have precipitated in different orientations can be obtained. In reference to previous studies and related literature^23,24^, Mg-Zn and Mg-Sn alloys with different secondary-phase morphologies were selected for investigation in this study.

The present study was designed to investigate the effect of secondary-phase orientation on the electromagnetic shielding properties of Mg-Zn and Mg-Sn alloys. This investigation will provide an important basis for the development of high-performance metallic materials for shielding applications and broaden the practical application of magnesium alloys in the electromagnetic compatibility field.

## Results and Discussion

### Microstructures

**Figure 1:**
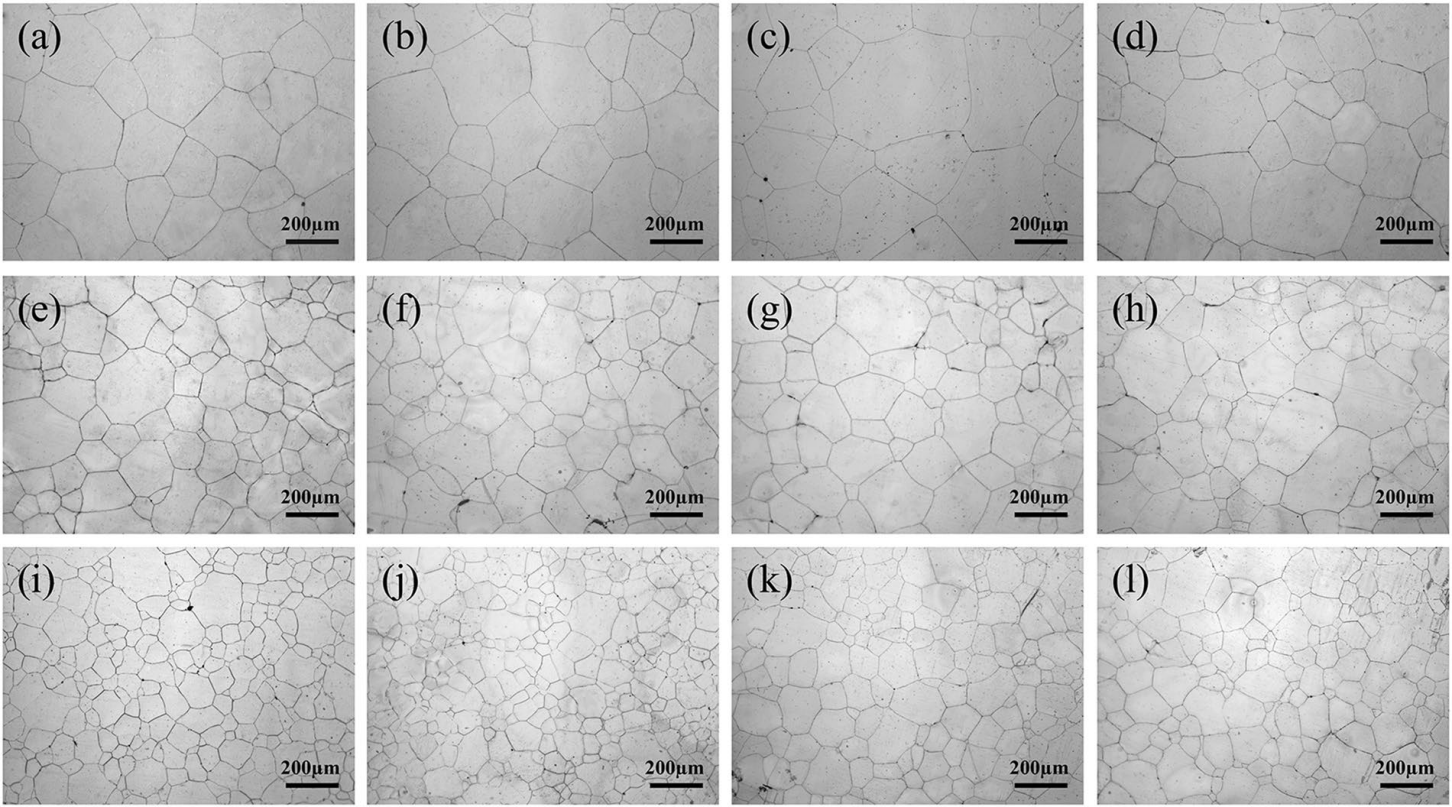
Optical microscopy images of Mg-3Zn alloy microstructures: (**a**) unrolled and subjected to solution treatment at 420 °C for 24 h; (**b**) further aged at 170 °C for 10 h, (**c**) 25 h, and (**d**) 60 h; (**e**) as-rolled (20%) and subjected to solution treatment at 420 °C for 24 h; (**f**) further aged at 170 °C for 10 h, (**g**) 25 h, and (**h**) 60 h; (**i**) as-rolled (50%) and subjected to solution treatment at 420 °C for 24 h; and (**j**) further aged at 170 °C for 10 h, (**k**) 25 h, and (**l**) 60 h.

**Figure 2:**
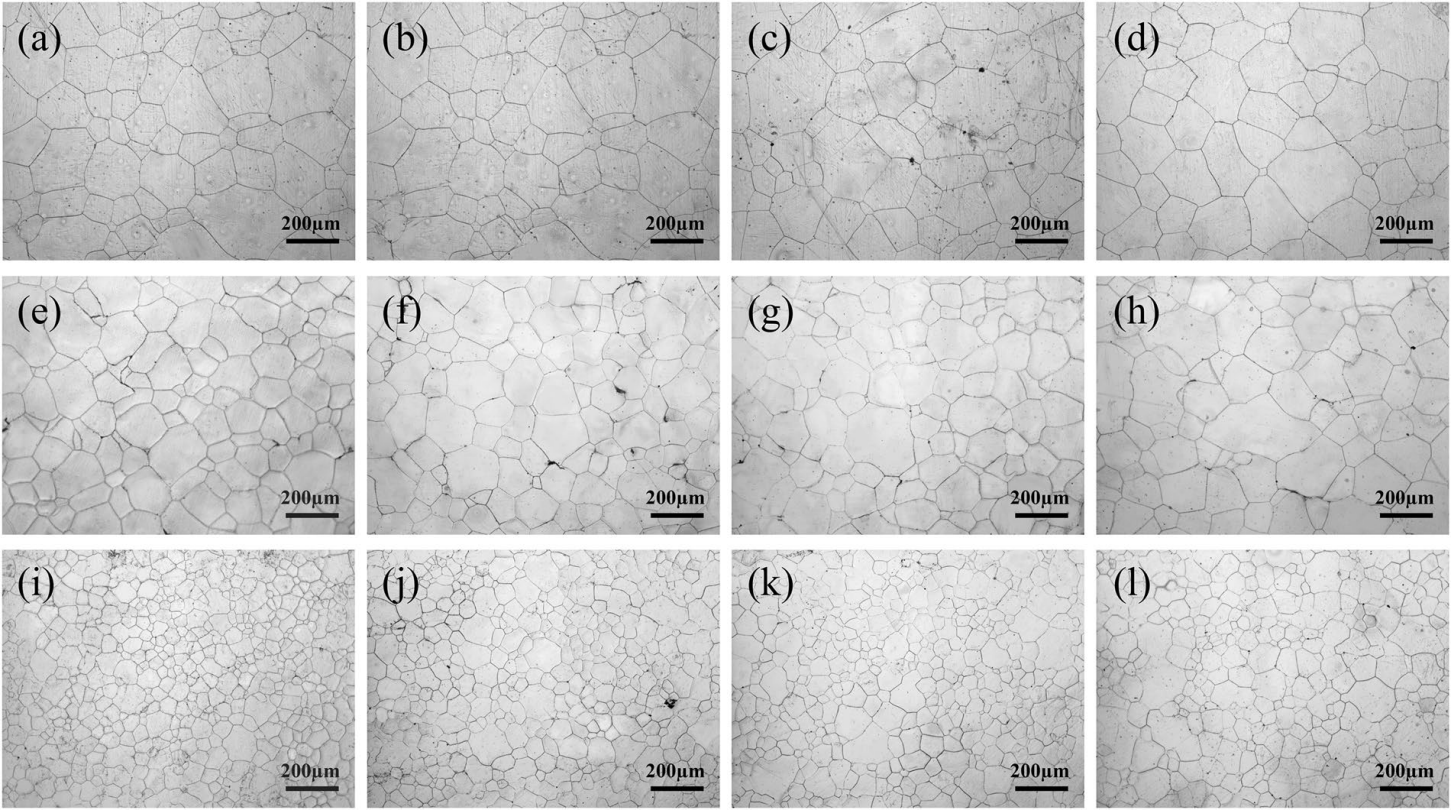
Optical microscopy images of Mg-5Zn alloy microstructures: (**a**) unrolled and subjected to solution treatment at 420 °C for 24 h, (**b**) further aged at 170 °C for 10 h, (**c**) 25 h, and (**d**) 60 h; (**e**) as-rolled (20%) and subjected to solution treatment at 420 °C for 24 h, (**f**) further aged at 170 °C for 10 h, (**g**) 25 h, and (**h**) 60 h; and (**i**) as-rolled (50%) and subjected to solution treatment at 420 °C for 24 h, (**j**) further aged at 170 °C for 10 h, (**k**) 25 h, and (**l**) 60 h.

**Figure 3:**
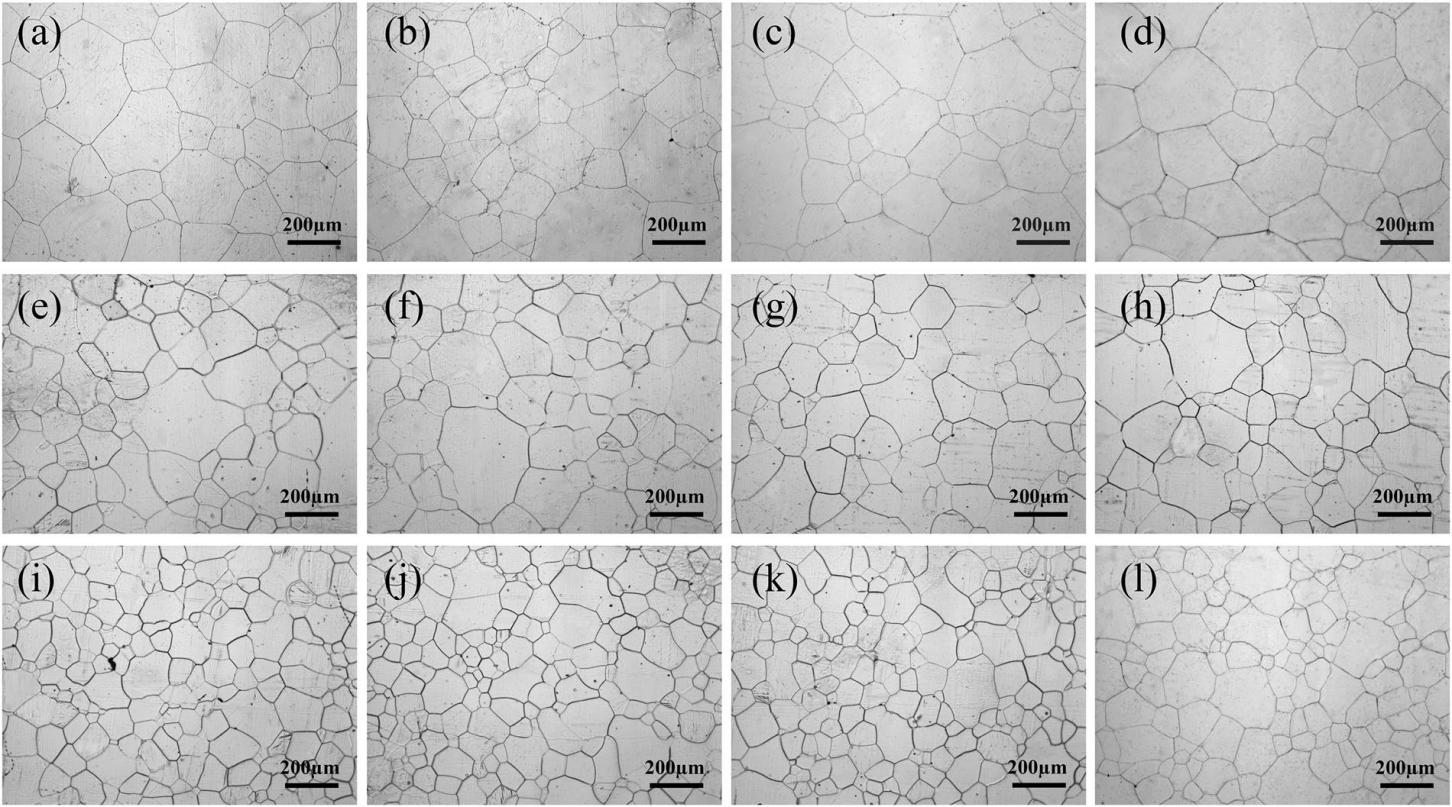
Optical microscopy images of Mg-3Sn alloy microstructures: (**a**) unrolled and subjected to solution treatment at 480 °C for 24 h, (**b**) further aged at 170 °C for 10 h, (**c**) 35 h, and (**d**) 60 h; (**e**) as-rolled (20%) and subjected to solution treatment at 480 °C for 24 h, (**f**) further aged at 170 °C for 10 h, (**g**) 35 h, and (**h**) 60 h; and (**i**) as-rolled (50%) and subjected to solution treatment at 480 °C for 24 h, (**j**) further aged at 170 °C for 10 h, (**k**) 35 h, and (**l**) 60 h.

**Figure 4:**
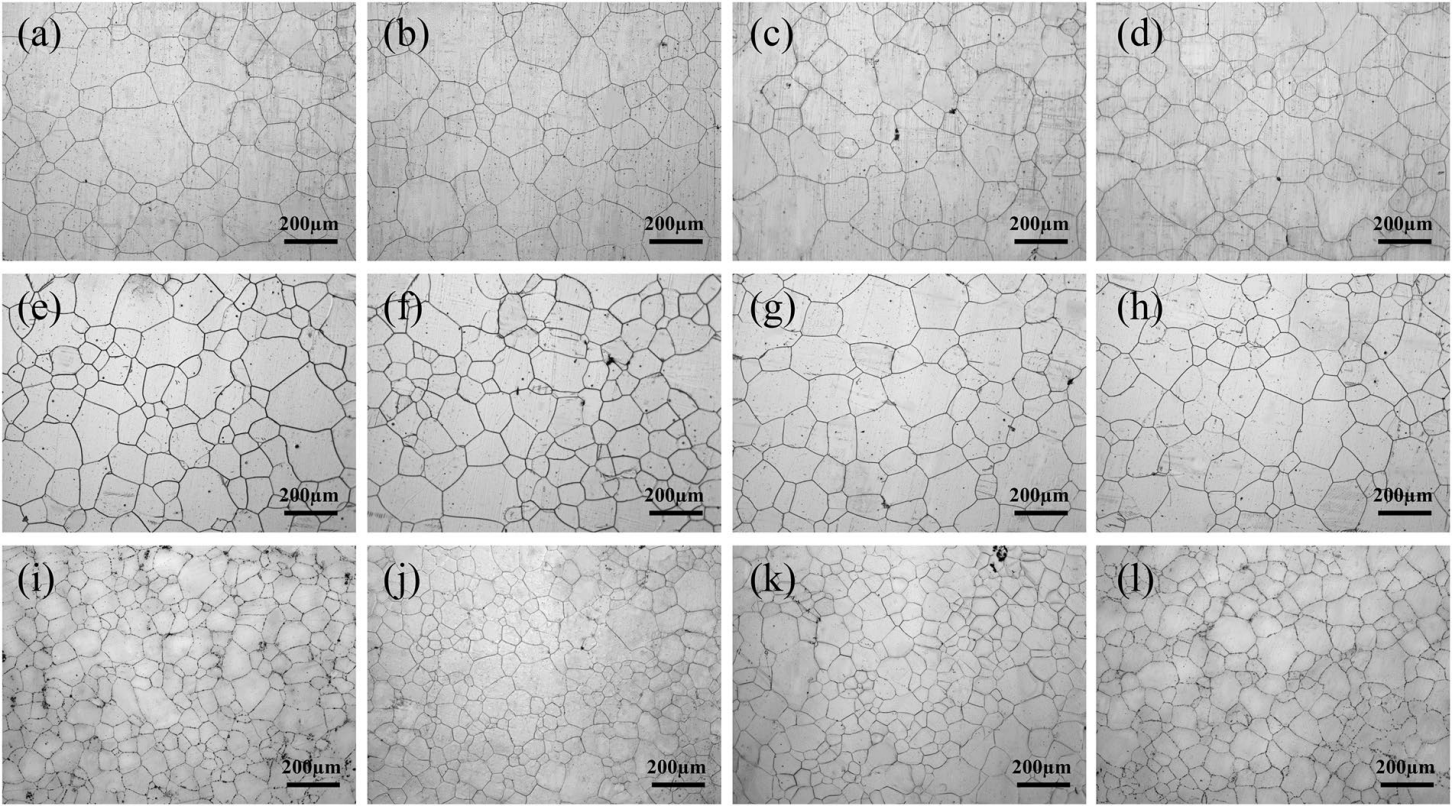
Optical microscopy images of Mg-5Sn alloy microstructures: (**a**) unrolled and subjected to solution treatment at 480 °C for 24 h, (**b**) further aged at 170 °C for 10 h, (**c**) 35 h, and (**d**) 60 h; (**e**) as-rolled (20%) and subjected to solution treatment at 480 °C for 24 h, (**f**) further aged at 170 °C for 10 h, (**g**) 35 h, and (**h**) 60 h; and (**i**) as-rolled (50%) and subjected to solution treatment at 480 °C for 24 h, (**j**) further aged at 170 °C for 10 h, (**k**) 35 h, and (**l**) 60 h.

Figures 1–4 show the optical microscopy images of the microstructures present in Mg-Zn and Mg-Sn alloys under different states. The samples exhibit a completely recrystallized structure after a long duration of solid-solution treatment, and the microstructures are composed of equiaxial grains with relatively uniform size. Compared with the microstructures of unrolled structures, those of rolled structures exhibit grain refinement. Grain size slightly increases as aging time is extended. The grain sizes of Mg-5Zn and Mg-5Sn alloys are finer than those of Mg-3Zn and Mg-3Sn alloys because the precipitation of the secondary phase restrains grain growth.

**Figure 5:**
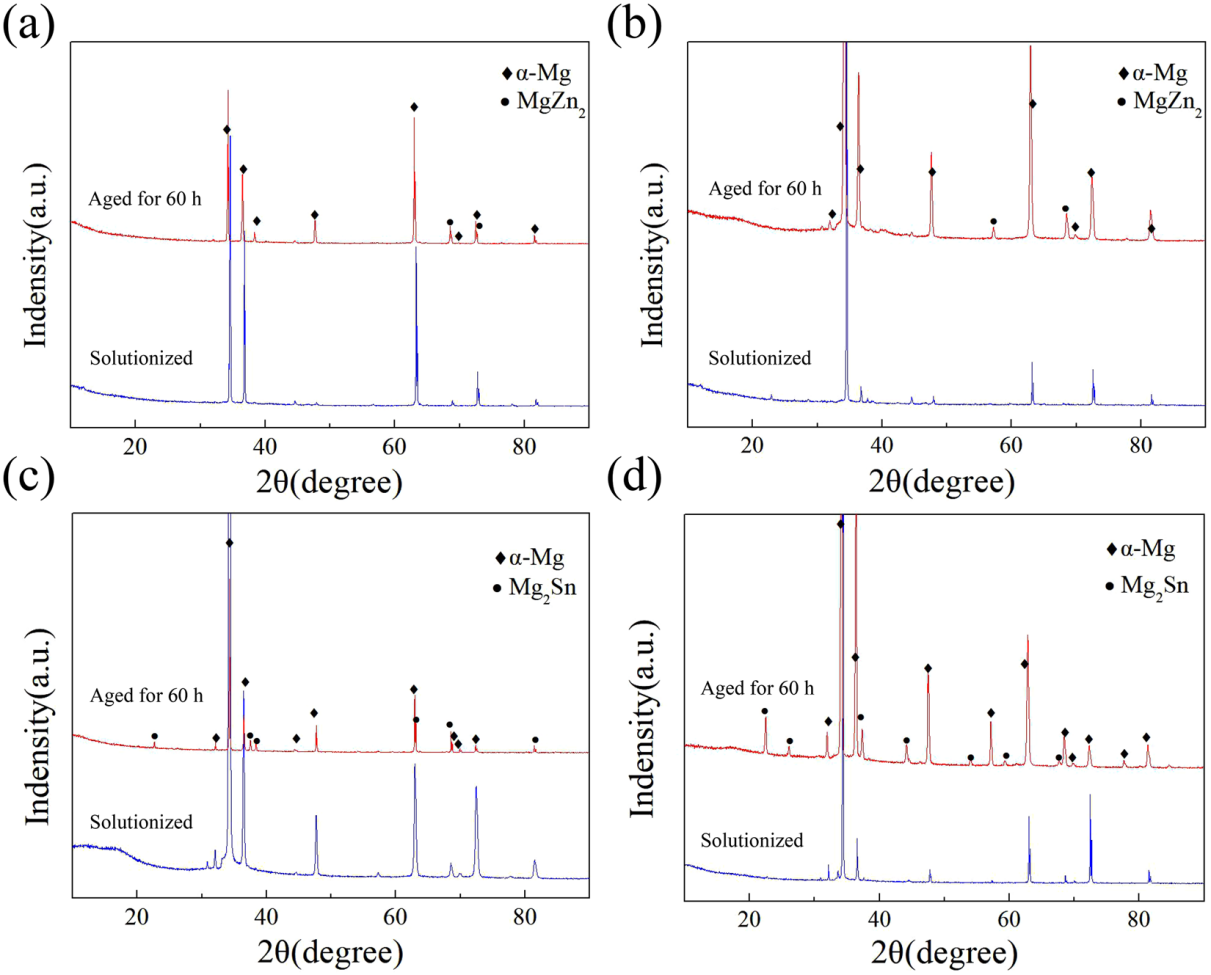
XRD patterns of solutionized and aged for 60 h after rolling (**a**) Mg-3Zn, (**b**) Mg-5Zn, (**c**) Mg-3Sn, and (**d**) Mg-5Sn alloys.

Figure 5 displays the typical X-ray diffraction (XRD) patterns of as-rolled Mg-Zn and Mg-Sn alloys in various states. Only peaks that correspond to the α-Mg matrix phase are present in the XRD patterns of the solutionized Mg-3Zn and Mg-5Zn samples, whereas peaks that correspond to MgZn_2_ phases can be clearly identified in the aged samples. The XRD patterns of Mg-5Sn alloys indicate that the aged samples mainly consist of α-Mg and Mg_2_Sn phases, whereas only α-Mg peaks are identified in the solutionized Mg-5Sn alloy. Therefore, most secondary phases dissolve in the α-Mg matrix, and the alloys are transformed into a solid solution. By contrast, during aging, the secondary phase precipitates from the α-Mg matrix.

**Figure 6:**
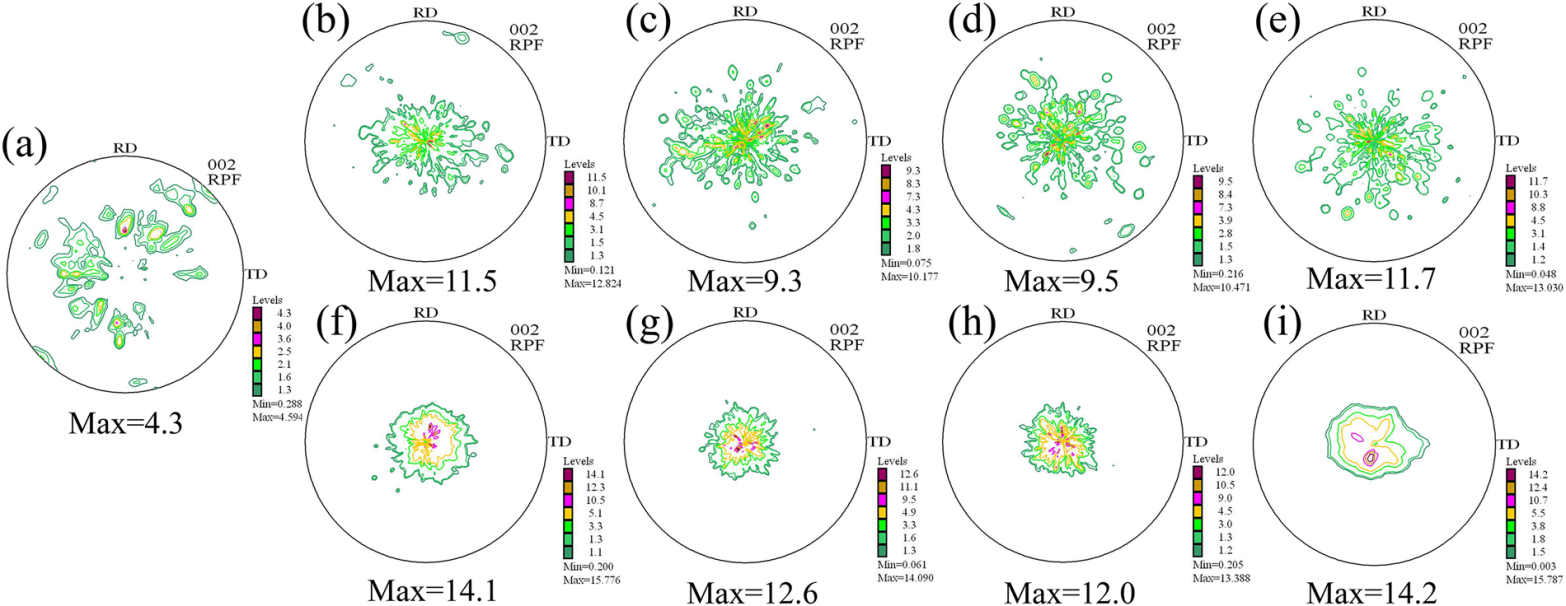
(0002) macrotextures of Mg-5Zn alloy in various states: (**a**) cast, (**b**) rolled (20%) and subjected to solution treatment at 420 °C for 24 h; (**c**) further aged at 170 °C for 10 h, (**d**) 25 h, and (**e**) 60 h; and (**f**) rolled (50%) and subjected to solution treatment at 420 °C for 24 h and (**g**) further aged at 170 °C for 10 h, (**h**) 25 h, and (**i**) 60 h.

**Figure 7:**
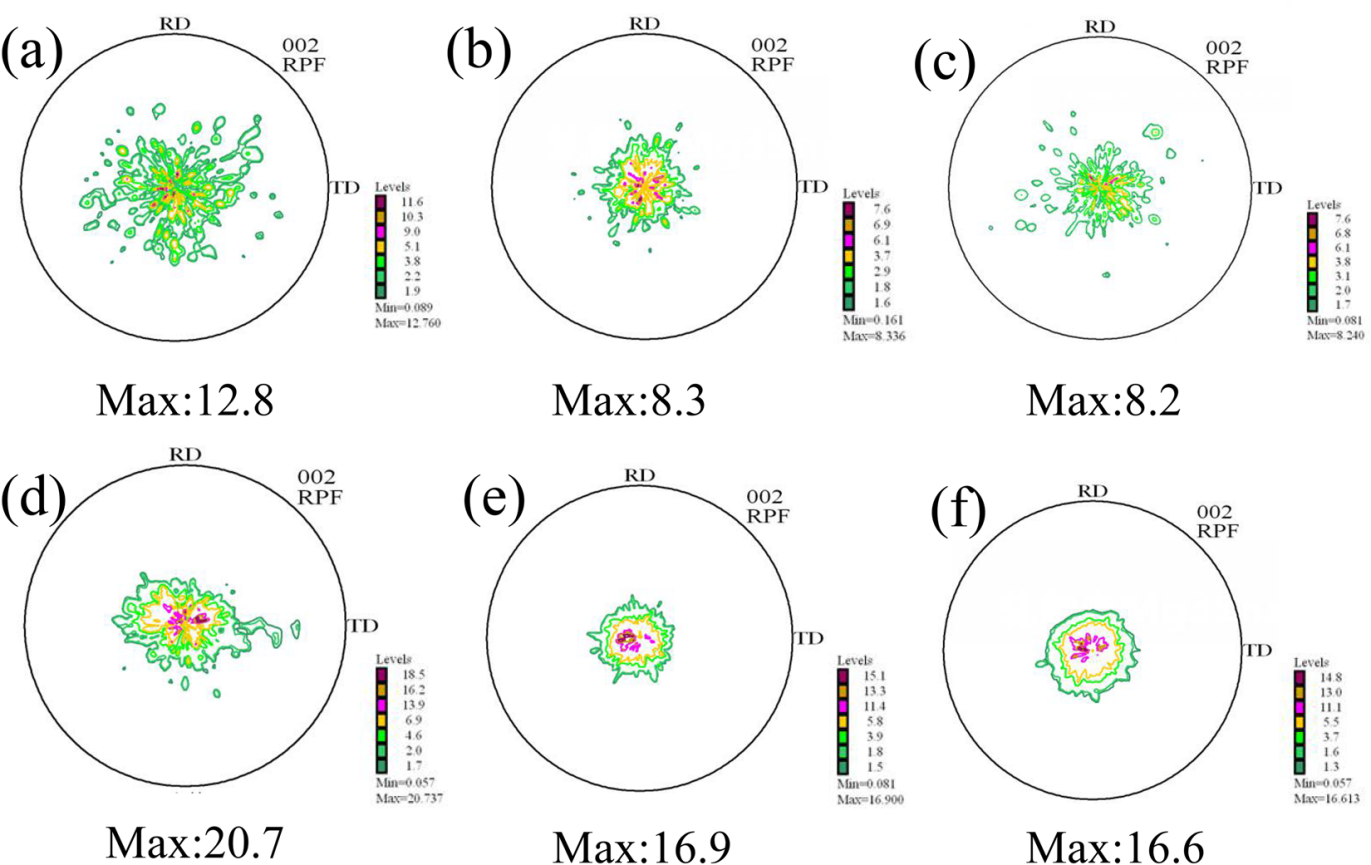
(0002) macrotextures of the studied alloys: (**a**) Mg-3Zn 20% rolled and aged for 25 h, (**b**) Mg-3Sn 20% rolled and aged for 35 h, (**c**) Mg-5Sn 20% rolled and aged for 35 h, (**d**)Mg-3Zn 50% rolled and aged for 25 h, (**e**) Mg-3Sn 50% rolled and aged for 35 h, and (**f**) Mg-5Sn 20% rolled and aged for 35 h.

**Figure 8:**
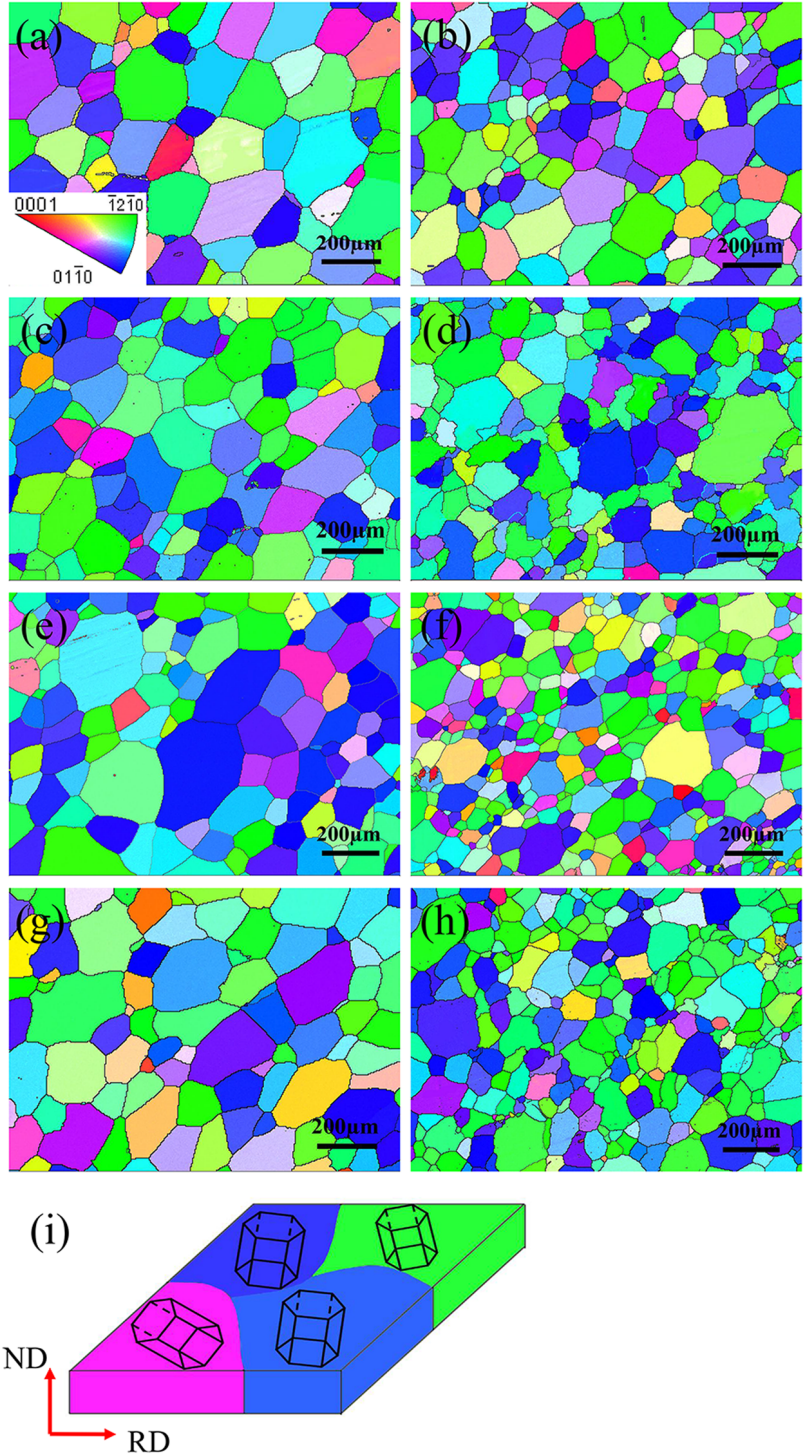
IPF images of the studied alloys: (**a**) Mg-3Zn 20% rolled and (**b**) 50% rolled aged for 25 h; (**c**) Mg-5Zn 20% rolled and (**d**) 50% rolled aged for 25 h; (**e**) Mg-3Sn 20% rolled and (**f**) 50% rolled aged for 35 h; (**g**) Mg-5Sn 20% rolled and (**h**) 50% rolled aged for 35 h; and (**i**) schematic of the grain orientation and microstructure of the alloy.

Figure 6 depicts the (0002) pole images of Mg-5Zn alloys in different states. As shown in Fig. 6a, the unrolled sample does not exhibit texture, and the grains of the alloy are randomly oriented. After rolling deformation, the texture of rolled samples changes from random to (0001) basal, and the maximum texture intensity also increases as rolling reduction increases. Meanwhile, grains in the alloy gradually rotate as the maximum texture intensity increases. This phenomenon indicates that c-axis of the grain deflects parallel to ND. Changes in texture type and intensity after aging are not distinct because the rolled sheet with large deformation has a strong basal texture before heat treatment, and most of the grains have already completely deflected to the [0001]_Mg_//ND direction. Therefore, extending aging time does not significantly change texture intensity. The variation trend of the texture of other alloys is similar to that of Mg-5Zn. (0002) pole images of 20% and 50% rolled Mg-3Zn alloys aged for 25 h, Mg-3Sn and Mg-5Sn alloys aged for 35 h are exhibited in Fig. 7. It can be clearly found that all the alloys exhibit significant basal texture, indicating that most of the grains are deflected. In order to further illustrate the orientation of grains, cross-sections from the samples were subjected to electron backscatter diffraction (EBSD) test for the direct observation of grain orientation. Figure 8a shows an IPF image of the Mg-3Zn alloy aged for 25 h. In this image, blue and green grains have c-axes parallel to ND, whereas red grains have c-axes parallel to TD. The IPF diagram shows that most of the grains are blue and green, indicating that the grains of the alloy have deflected to the direction in which the c-axis is parallel to ND and that only a few grains have deflected along TD. The grain orientation of other alloys is similar to that of Mg-3Zn alloys.

**Figure 9:**
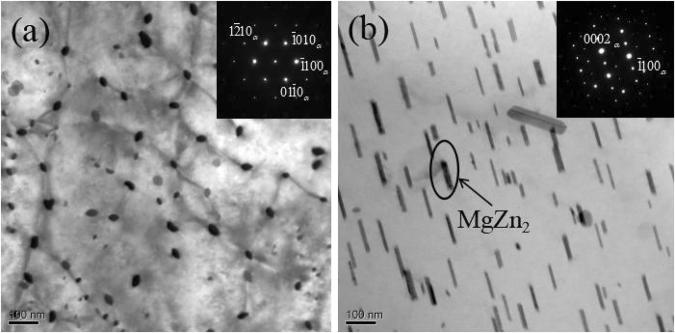
Bright-field TEM images of Mg-3Zn alloys in the (**a**) [0001] and (**b**) $[11\bar{2}0]$ direction of α-Mg.

The bright-field transmission electron microscopy (TEM) images of Mg-3Zn alloy aged for 60 h and observed along the [0001]_Mg_ and ${[11\bar{2}0]}_{{\rm{Mg}}}$ zone axes are shown in Fig. 9. Precipitates are dispersed and evenly formed on the basal plane (Fig. 9a). In Fig. 9b, precipitates are present as a rod-like phase that is uniformly distributed over the α-Mg matrix. The growth axes of the precipitates are parallel to the [0001]_Mg_ direction. All precipitates are similarly oriented within a grain. Energy-dispersive X-ray spectroscopy (EDS) analyses through conventional TEM indicated that the precipitates contain Zn and Mg. The distribution of precipitates in Mg-5Zn alloy is similar to that in Mg-3Zn alloy. Referring to previous studies and related literature^22,23^, the precipitated phase is identified as MgZn_2_ phase (${\beta ^{\prime} }_{1}$-phase). Its long-axis direction is parallel to [0001]_Mg_. The phase relation of the MgZn_2_ phase and α-Mg matrix is as follows:

$${(0001)}_{\beta }//{(0001)}_{{\rm{Mg}}},\,{[11\overline{2}0]}_{\beta }//{[10\overline{1}0]}_{{\rm{Mg}}}.$$

**Figure 10:**
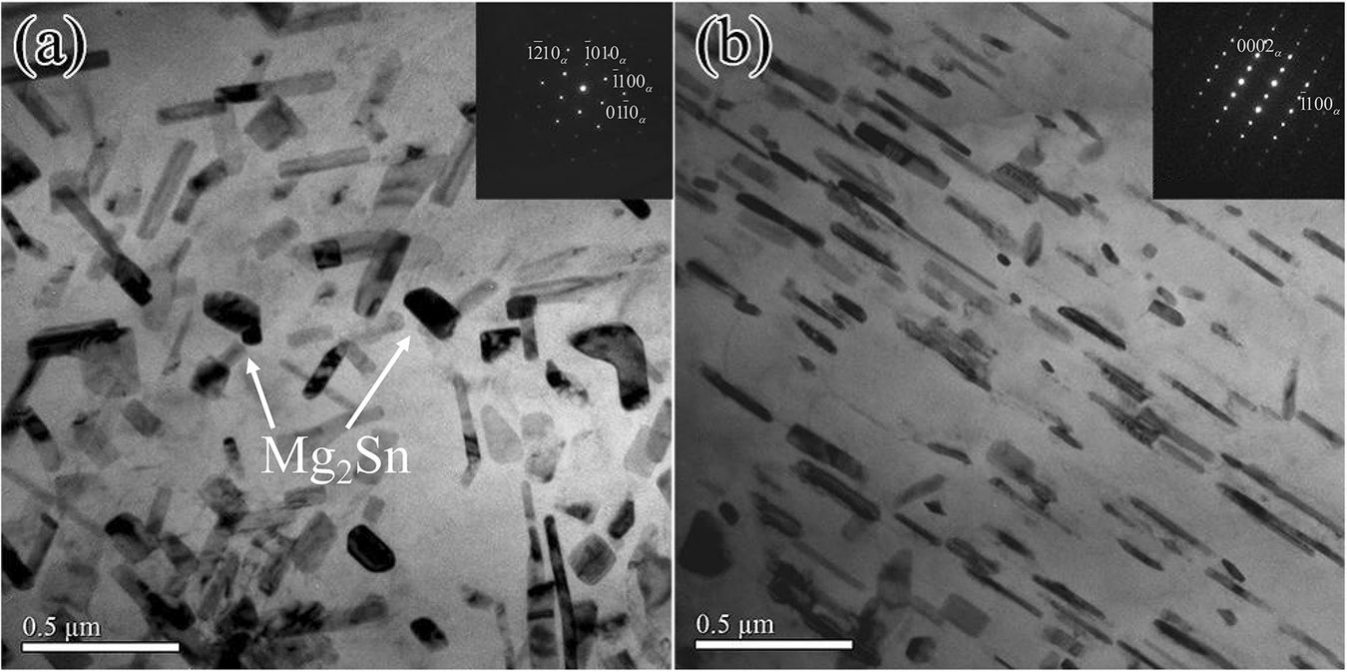
Bright-field TEM images of Mg-3Sn alloys in the (**a**) [0001] and (**b**) $[11\bar{2}0]$ direction of α-Mg.

Figure 10 shows the bright-field TEM images of Mg-3Sn alloy aged for 60 h and observed along the [0001]_Mg_ and ${[11\bar{2}0]}_{{\rm{Mg}}}$ zone axes. Plate-like precipitates are randomly and densely distributed on the basal plane of the α-Mg matrix (Fig. 10a). The precipitates appear as small rod-like particles with similar orientations (Fig. 10b). EDS analyses indicated that the precipitates contain Sn and Mg. The distribution of precipitates in the Mg-5Sn alloy is similar to that in the Mg-3Sn alloy. By referring to previous studies and related literature^24^, the secondary phase is identified as the Mg_2_Sn phase, and the phase relation of the Mg_2_Sn phase and the α-Mg matrix is as follows:

$${(0001)}_{{\rm{Mg}}}//{(0\overline{3}\overline{3})}_{{{\rm{Mg}}}_{2}{\rm{Sn}}},\,{[2\overline{1}\overline{1}0]}_{{\rm{Mg}}}//{[\overline{1}22]}_{{{\rm{Mg}}}_{2}{\rm{Sn}}}$$

### Electrical conductivity

**Figure 11:**
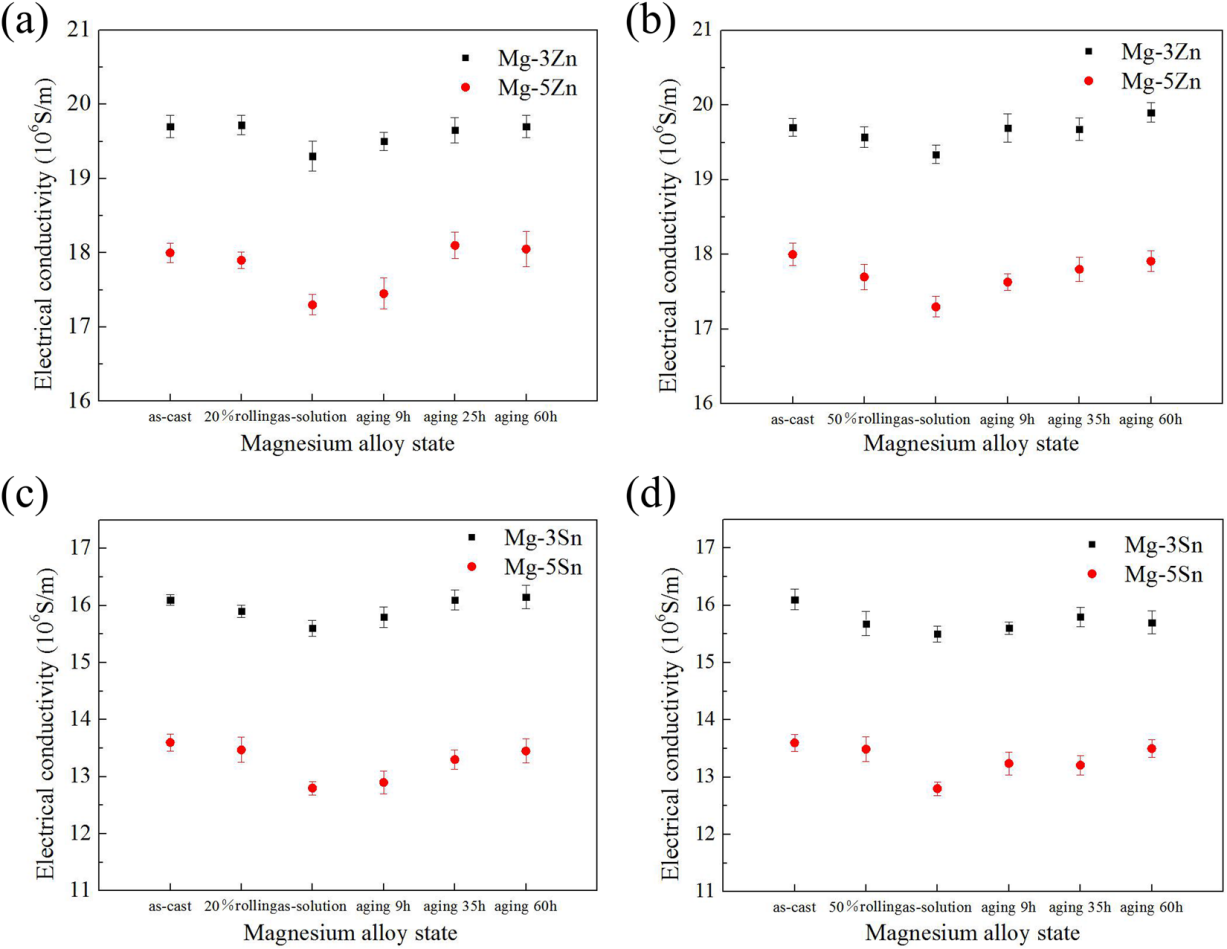
Electrical conductivity of the alloys in different states: (**a**,**c**) 20% rolled and (**b**,**d**) 50% rolled.

The electrical conductivity of Mg-2Zn, Mg-5Zn, Mg-3Sn, and Mg-5Sn alloys in different states at room temperature are summarized in Fig. 11. After solution treatment, the electrical conductivities of Mg-2Zn and Mg-5Zn alloys have slightly decreased by 0.4 and 0.7 MS/m, respectively. Extending aging time from 10 h to 25 h increases the electrical conductivity of Mg-3Zn alloy by 0.35 MS/m, which is lower than the 0.8 MS/m increment for the electrical conductivity of Mg-5Zn alloy. Prolonging aging time to 60 h negligibly changes conductivity. The variation trend of the electrical conductivity of Mg-Sn alloy is similar to that of the electrical conductivity of Mg-Zn alloy. In addition, the electrical conductivity of Mg-Sn alloy is lower than that of Mg-Zn alloy.

The Matthiessen criterion^25,26^ states that the resistivity (*ρ*) of metals, which is the reciprocal of conductivity, can be obtained using the following formula:

1 $${\rho }={{\rho }}_{{Mg}}+{\rho }\text{'}+{\rm{\Delta }}$$

2 $${\rho }\text{'}={C}{\rm{\Delta }}{\rho }$$

where *ρ*_*Mg*_, C, Δ*ρ*, and Δ are the resistivity of pure magnesium, content of solute atoms, excess resistivity attributed to 1% solute atoms, and deviations from the value of Matthiessen’s law, respectively. Notably, the resistivity of pure magnesium is only influenced by electron–phonon interactions that are caused by lattice distortion induced by thermal excitation. In addition, the effect of microstructural defects (vacancies, dislocation, twinning, and grain boundary) on resistivity in the matrix is negligible and can be ignored. Hence, at ambient temperature, solute atoms play a major role in resistivity. Solid-solution treatment can dissolve the secondary phase into the matrix, and solute atoms dissolved in matrix will cause crystal lattice distortion, which has a serious scattering effect on the electron transfer process. These effects collectively increase resistivity. The increase in alloy resistivity caused by the dissolution of alloying elements in the matrix is approximately a magnitude order higher than that caused by the precipitation of alloying elements^27^. Therefore, solution treatment considerably decreases the conductivities of Mg-Zn and Mg-Sn alloys. Alloy elements precipitate from the matrix with extended aging time. This effect decreases lattice distortion, consequently increasing electrical conductivity.

### Shielding effectiveness

**Figure 12:**
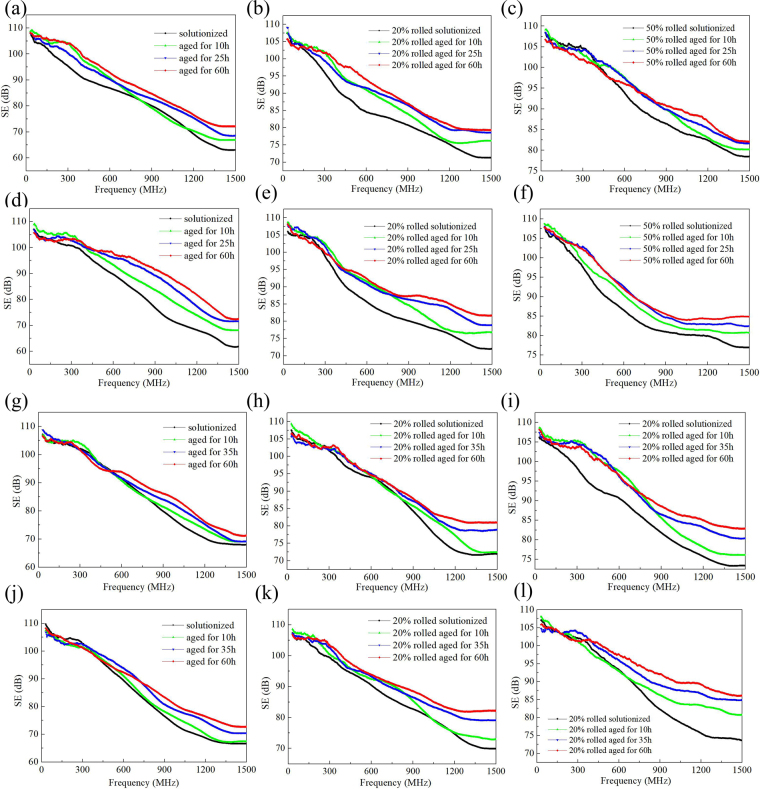
Variation in electromagnetic shielding effectiveness with frequency: (**a**–**c**) Mg-3Zn, (**d**–**f**) Mg-5Zn, (**g**–**i**) Mg-3Sn, and (**j**–**l**) Mg-5Sn.

The frequency dependences of the EMI SE of Mg-3Zn, Mg-5Zn, Mg-3Sn, and Mg-5Sn alloys in different states are illustrated in Fig. 12. In the frequency range of 30–1500 MHz, the SE value decreases monotonically with increasing test frequency. The SE value shows no obvious change in the range of 30–300 MHz after aging but gradually increases in the range of 300–1500 MHz with extended aging time. The SE values of the unrolled Mg-3Zn, Mg-5Zn, Mg-3Sn, and Mg-5Sn alloys increase by 10, 13, 4, and 8 dB at 1200 MHz as they transition from a solid-solution state to the 35 h-aged state, respectively. The variation trend of the SE value basically corresponds to the monotonic change in electrical conductivity. With increasing rolling reduction, the EMI SE increases gradually. For example, the SE value of solutionized Mg-3Zn alloy increases by 9.8 and 14.5 dB at 1200 MHz after rolling with 20% and 50% reduction. For most alloys, when aging is prolonged to 60 h, the promotion of SE value is not evident.

**Figure 13:**
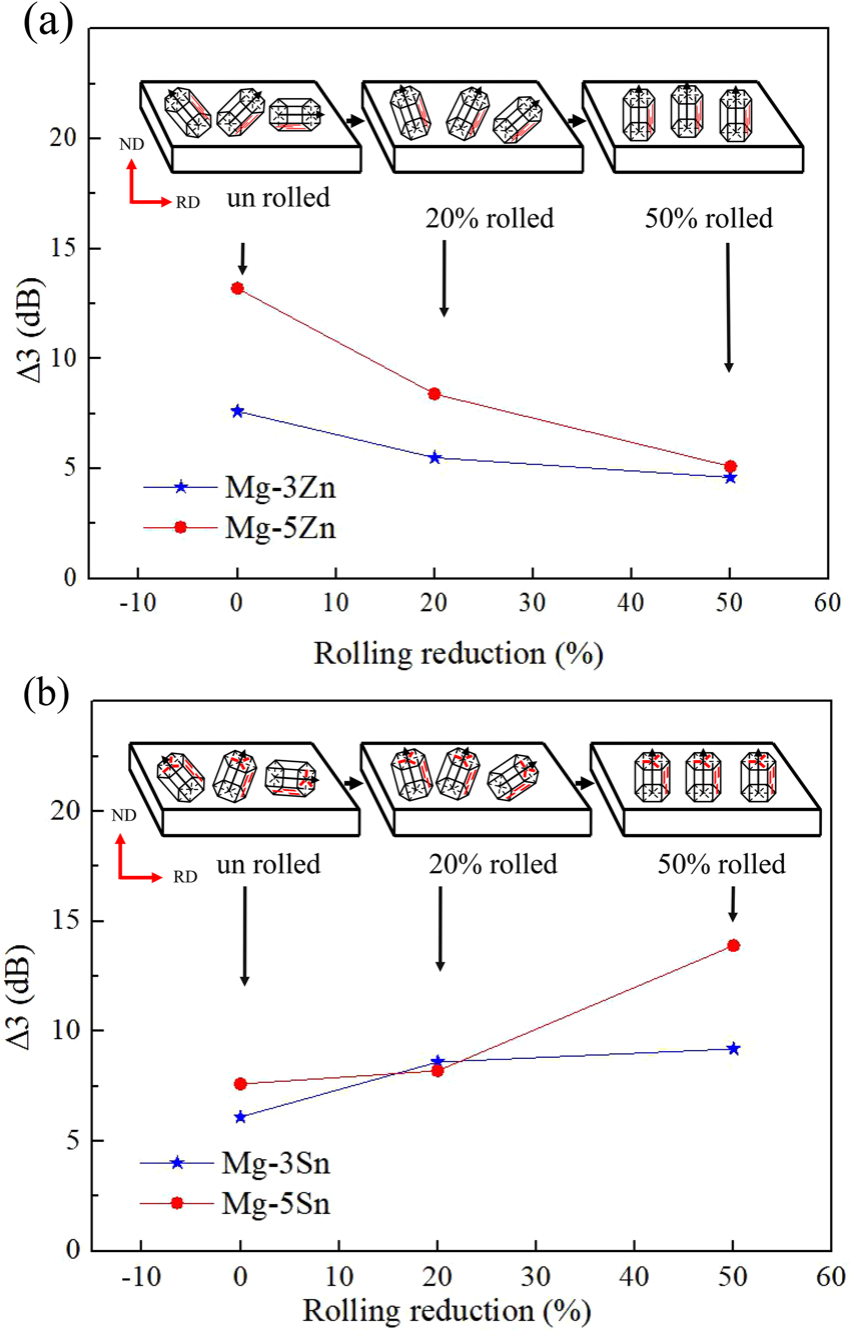
Effect of secondary-phase orientation on the electromagnetic shielding properties of (**a**) Mg-Zn and (**b**) Mg-Sn alloys (Δ3 = SE_aged (60 h)_–SE_solutionized_).

To further investigate the change in SE caused by the orientation of secondary-phase precipitates, the influence of texture must be removed because the change in secondary-phase orientation is accompanied by a change in texture. Therefore, the effect of secondary-phase orientation on EMI SE is characterized on the basis of the difference in SE values between aging and solid solution states at 1200 MHz. Tables 1–4 present the increments in the SE value of Mg-Zn and Mg-Sn alloys when transitioning from a solid-solution state to an aged state. For Mg-Zn alloy, the increment in SE value decreases gradually with increasing rolling reduction. For example, for unrolled Mg-5Zn alloy, the difference in SE values between samples in solid-solution state and 60 h-aged state is 13.2 dB and those of 20% and 50% rolled Mg-5Zn alloy decrease to 8.4 and 5.1 dB, respectively. The trend of the EMI SE of Mg-Sn alloy opposes that of the Mg-Zn alloy. Specifically, the EMI SE of Mg-Sn alloy increases as rolling deformation increases. With Mg-5Sn alloy as an example, the SE value of unrolled sample increases by 7.6 dB from solid-solution state to 60 h-aged state, whereas the added SE values of 20% and 50% rolled samples increase to 8.2 and 13.9 dB, respectively. Figure 13 presents a comparison of the effects of secondary phases with different orientations on the electromagnetic shielding properties of Mg-Zn and Mg-Sn alloys. The plate-like Mg_2_Sn phase that has precipitated on the (0001) basal plane is more beneficial for improving the electromagnetic shielding properties of the alloy than the rod-like MgZn_2_ phase that has precipitated along the $[11\bar{2}0]$ plane.

**Table 1:** EMI shielding effectiveness of Mg-3Zn alloy in various states.

Alloys	Shielding effectiveness at 1200 MHz (dB)				Δ1	Δ2	Δ3
	Solutionized	Aged (10 h)	Aged (25 h)	Aged (60 h)			
3Zn-0%	68.9	70.2	75.2	76.5	1.3	6.3	7.6
3Zn-20%	75.2	75.9	79.5	80.7	0.7	4.3	5.5
3Zn-50%	82.4	82.9	85.2	86.8	0.5	2.8	4.6

^*^Δ1 = aged (10 h)–solutionized, Δ2 = aged (25 h)–solutionized, Δ3 = aged (60 h)–solutionized.

**Table 2:** EMI shielding effectiveness of Mg-5Zn alloy in various states.

Alloys	Shielding effectiveness at 1200 MHz (dB)				Δ1	Δ2	Δ3
	Solutionized	Aged (10 h)	Aged (25 h)	Aged (60 h)			
5Zn-0%	68.2	73.8	77.0	81.4	5.6	8.8	13.2
5Zn-20%	76.1	77.2	83.4	84.5	1.1	7.3	8.4
5Zn-50%	79.8	81.4	82.9	84.9	1.6	3.1	5.1

^*^Δ1 = aged (10 h)–solutionized, Δ2 = aged (25 h)–solutionized, Δ3 = aged (60 h)–solutionized.

**Table 3:** EMI shielding effectiveness of Mg-3Sn alloy in various states.

Alloys	Shielding effectiveness at 1200 MHz (dB)				Δ1	Δ2	Δ3
	Solutionized	Aged (10 h)	Aged (35 h)	Aged (60 h)			
3Sn-0%	70.1	73.5	75.2	76.2	3.4	5.1	6.1
3Sn-20%	72.9	77.1	79.2	81.5	4.2	6.3	8.6
3Sn-50%	75.7	78.1	83.1	84.9	2.4	7.4	9.2

*Δ1 = aged (10 h)–solutionized, Δ2 = aged (25 h)–solutionized, Δ3 = aged (60 h)–solutionized.

**Table 4:** EMI shielding effectiveness of Mg-5Sn alloy in various states.

Alloys	Shielding effectiveness at 1200 MHz (dB)				Δ1	Δ2	Δ3
	Solutionized	Aged (10 h)	Aged (35 h)	Aged (60 h)			
5Sn-0%	68.7	70.1	74.6	76.3	1.4	5.9	7.6
5Sn-20%	74.3	76.2	80.8	82.5	1.9	6.5	8.2
5Sn-50%	75.3	83.3	86.6	89.2	8	11.3	13.9

*Δ1 = aged (10 h)–solutionized, Δ2 = aged (25 h)–solutionized, Δ3 = aged (60 h)–solutionized.

The loss experienced by an electromagnetic wave transmitted through a shielding material can be divided into (1) reflection loss (*R*) at the two interfaces of the shielding material, (2) absorption loss (*A*) in the body of the shielding material, and (3) multiple reflection loss (*B*) in the body of shielding material^8,10^. For a plain-wave electromagnetic radiation, the total electromagnetic SE can be expressed as the following equation^28,29^:

3 $${SE}(dB)={R}+{A}+{B}$$

4 $${S}{{E}}_{{R}}=20{\mathrm{log}}_{10}\frac{{|1+{k}|}^{2}}{4|{k}|},({k}={{Z}}_{{s}}/{{Z}}_{0})$$

5 $$S{E}_{A}=20{\mathrm{log}}_{10}({e}^{{t}_{s}/{\delta }_{s}})$$

6 $$S{E}_{B}=20{\mathrm{log}}_{10}|1-\frac{{({\rm{k}}-1)}^{2}}{{(k+1)}^{2}}{e}^{\frac{-2{t}_{s}}{{\delta }_{s}}}|$$

7 $${Z}_{s}=\sqrt{j\omega {\mu }_{s}/{\delta }_{s}}$$

8 $${\delta }_{s}=\sqrt{2/\omega {\mu }_{s}{\sigma }_{s}},$$

where *Z*_*s*_, *Z*_0_, *δ*_*s*_, *ω*, *σ*_*s*_, *μ*_*s*_, and *t*_*s*_ are the shield impedance, air impedance (377 Ω), skin depth, electromagnetic radiation frequency, electrical conductivity, magnetic permeability, and thickness, respectively. On the basis of Eqs 4 and 5, the increase in electrical conductivity would promote interaction between free electrons and the electromagnetic field; this interaction can enhance *SE*_R_. Moreover, the heat loss caused by the interaction would increase *SE*_A_^16^. Therefore, the variation tendency of the electromagnetic shielding performance of magnesium alloys in different states is similar to that of electrical conductivity. *SE*_*R*_ decreases and *SE*_*A*_ improves as the frequency of the incident electromagnetic wave increases. For a metal shield to be considered as a good conductor, its *SE*_*R*_ should be considerably higher than its *SE*_*A*_. As a result, SE decreases monotonically when the frequency of the incident electromagnetic wave increases. However, the secondary phase precipitated on the matrix during aging would affect the electromagnetic shielding properties of the alloy in addition to the above factors.

During aging, texture does not markedly change, and grain size has a negligible effect on electromagnetic shielding performance^14^; hence, the secondary phase that precipitates during aging has a major influence on the EMI *SE* of alloys. With the extension of aging time, additional secondary phases precipitate, and the distribution of secondary phases become dense and uniform. When densely distributed additional secondary phases are gathered together continuously, the α-Mg matrix can be effectively separated and the discontinuity of the internal alloy structure are added. As a result, more interfaces are provided for the reflection of electromagnetic waves. These effects help prevent the transmission of electromagnetic waves in the alloy and enhance multiple reflection loss in the alloy.

**Figure 14:**
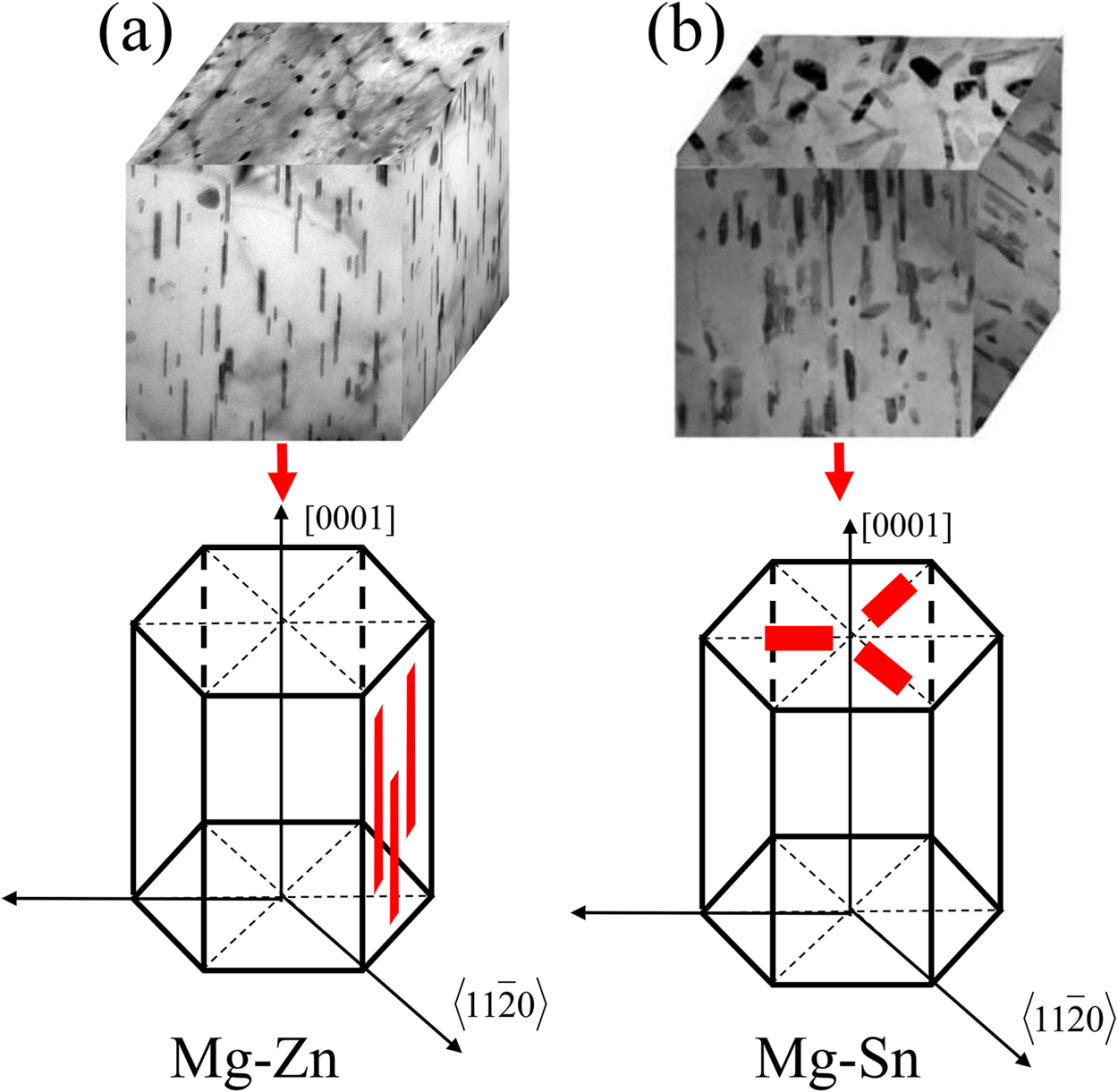
Schematic of secondary-phase orientation: (**a**) Mg-Zn and (**b**) Mg-Sn.

In addition to the change in number and distribution of the secondary phase in aging, the orientation of the secondary phase will also affect the SE of the alloy. The basal texture of the alloy is enhanced after rolling deformation. Grains tend to obtain a regular arrangement and are deflected to the direction in which the c-axis gradually becomes parallel to the ND. The secondary phase precipitates in a specific direction during aging, and grain orientation can be controlled by rolling. Thus, the orientation of the secondary phase, which is precipitated along a specific crystal plane, can be controlled. For Mg-Sn and Mg-Zn alloys, most of the Mg_2_Sn particles that precipitate on the basal plane of the matrix are plate-like in morphology, whereas most of the MgZn_2_ particles that precipitate with their growth axis parallel to the [0001]_Mg_ direction are rod-like in morphology. After rolling, the phase that precipitates during aging also has a certain orientation, and the rod-shaped phase is parallel to the incident direction of the electromagnetic wave. By contrast, the Mg_2_Sn phase precipitates in the plane perpendicular to the incident direction of the electromagnetic wave. A schematic of the different types of precipitates is presented in Fig. 14.

**Figure 15:**
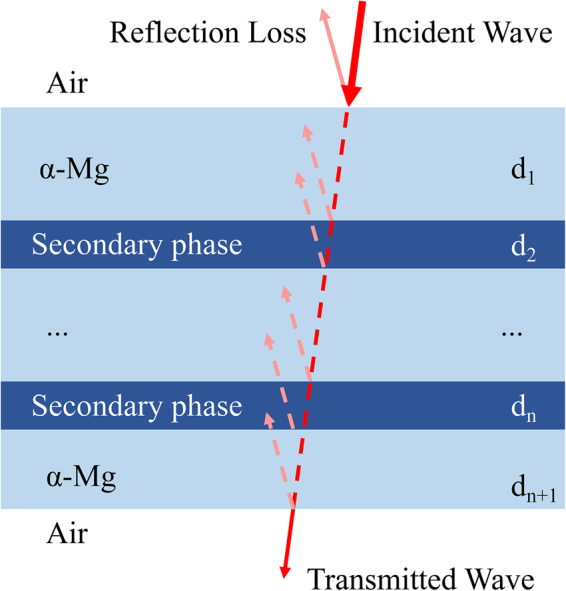
Double-layer shield model^28^.

Here, the morphology of the secondary phase may be idealized. A large number of densely distributed secondary phase can be seen as a coherent whole. The plate-like Mg_2_Sn phase forms an infinite plate along the basal plane, and the rod-like MgZn_2_ phase becomes an infinite slab along the prismatic plane. The schematic of this structure is shown in Fig. 15. In the alloy, the secondary phase forms a gap layer *d*_n_, and two sides of the gap layer are α-Mg. This structure constitutes a basic double-layer shield model, and the magnesium alloy can be considered as a combination of numerous double-layer shield models^28^.

The impedances of air, α-Mg matrix, and secondary phase are denoted as *Z*_0_, *Z*_s_, and *Z*_p_, respectively. The electrical conductivity of the secondary phase is extremely poor and is two orders of magnitude lower than that of pure magnesium^18^. In this case, the secondary phase can be regarded as an insulator; therefore, *Z*_p_ = *Z*_0_ = 377 Ω ≫ *Z*_s_^29^. Severe impedance mismatch occurs due to the large difference between the impedance of the secondary phase and α-Mg matrix. Eq. 3 states that multireflection loss is more likely to occur when the electromagnetic wave transmits through the interfaces between α-Mg and gap layer *d*. Moreover, in a single double-layer shield model, the gap layer formed by the secondary phase separates the magnesium matrix; thus, two internal interfaces are added. Reflection loss will be generated twice when the electromagnetic wave transmits through the shield.

In rolled Mg-Zn alloys, most of these gap layers are parallel to the incident direction of the electromagnetic wave, and the number of gap layers that can effectively reflect the electromagnetic wave is less than that in the unrolled Mg-Zn alloy. Therefore, electromagnetic shielding performance cannot be suitably improved when the orientation of the secondary phase tends to be consistent. Most gap layers in the rolled Mg-Sn alloy are perpendicular to the incident direction of the electromagnetic wave. Thus, the rolled sheet can be regarded as a superposition of several double-layer shield models. As a result, reflection loss is significantly enhanced. The above discussion indicates that compared with the rod-like MgZn_2_ phase, the plate-like Mg_2_Sn phase that precipitates along the (0001) basal plane can more effectively prevent the transmission of the incident electromagnetic wave.

The present experimental results indicated that controlling the orientation of secondary-phase precipitates in magnesium alloys is an effective method for developing future high-performance EMI-shielding metallic materials.

## Materials and Methods

Alloys with nominal compositions of Mg-xZn and Mg-xSn (x = 3, 5 wt.%) were prepared through conventional casting with high-purity magnesium (99.98 wt.%), industrial-grade zinc (99.9 wt.%), and industrial-grade tin (99.9 wt.%) under the protective atmosphere of CO_2_ and SF_6_ (100:1). The ingots were homogenized at 380 °C for approximately 12 h. Wafers with dimensions of Φ133 × 2.5 mm and Φ133 × 5.1 mm were cut from the middle of the ingots. Rolling was conducted at a speed of 3.5 m/min. The reduction per pass was 10% throughout rolling until the sample was rolled into a plate with a thickness of 2 mm. The rolled Mg-Zn and Mg-Sn alloy samples were subjected to solution treatment for 24 h at 420 °C and 480 °C, respectively, and then aged at 170 °C for 10, 25, 35, and 60 h.

Microstructures were observed though optical microscopy (OM), EBSD (FEI Nova 400 FEG equipped with an HKL channel 5 systems), and TEM (Tecnai G2 F20 S-TWIN) with an accelerating voltage of 200 kV. For OM observation, the polished specimens were etched with a mixture comprising 1.5 g of picric acid, 25 ml of ethanol, 5 ml of acetic acid, and 10 ml of water. Thin foil specimens for TEM observation were prepared through mechanical polishing and ion-beam thinning using Gatan Precision Ion Polishing System at room temperature. Specimens for EBSD orientation mapping were prepared through mechanical grinding and electropolishing with an AC2 electrolyte solution. Phase and texture analyses were performed with a Rigaku D/MAX-2500PC XRD. The electrical conductivity of alloy specimens along ND under different conditions was measured with a conductivity meter (Sigmascope SMP 10) at ambient temperature. Each datum was the average of at least 10 testing results.

The EMI SE of the specimens was measured through the standard coaxial cable method in accordance with ASTM D4935-2010. The setup consisted of a DN 1015 A SE tester with its input and output connected to an Agilent 8753ES network analyzer, which is similar to that used in previous studies^30–32^. The test standard used for measuring EMI SE in the literature is usually ASTM D4935-99. Specimens in the form of discs 115 mm in diameter and 2 mm in thickness were prepared for EMI SE measurement and measured at the frequency range of 30–1500 MHz.

## Conclusions

Rolled Mg-Zn and Mg-Sn alloy sheets were subjected to solution treatment and aged. The EMI SE values of samples in different states were investigated to clarify the effect of secondary-phase orientation on the SE of magnesium alloys. The experimental results indicated that SE is improved when the secondary phase precipitates along the basal plane perpendicular to the incident direction of electromagnetic wave. The improvement in SE, however, is not as apparent when the secondary phase precipitates along other planes. This phenomenon indicates that the regular arrangement of Mg_2_Sn precipitates increases the number of available interfaces in Mg-Sn alloy that can reflect electromagnetic waves. Consequently, the enhancement in reflection and multiple reflection losses of the incident electromagnetic wave mainly contribute to the increment in SE. Mg-5Sn alloys exhibit the maximum increment in SE value of 13 dB at 1200 MHz after 16 h of solution treatment at 480 °C and 60 h of artificial aging at 170 °C.

### Data Availability

The datasets generated during and/or analyzed during the current study are available from the corresponding author upon reasonable request.
